# Early experiences and the development of emotional learning systems in rats

**DOI:** 10.1186/2045-5380-3-8

**Published:** 2013-04-10

**Authors:** Bridget L Callaghan, Rick Richardson

**Affiliations:** 1 School of Psychology, The University of New South Wales, Sydney, New South Wales, Australia

**Keywords:** Infantile amnesia, Extinction, Fear, Maternal-separation, Developmental trajectory, Emotion, Development

## Abstract

Research first reported nearly 50 years ago demonstrated that infant and young animals (including humans) exhibit profoundly faster rates of forgetting (i.e., infantile amnesia) than do adults. In addition to these differences in retention, more recent research has shown that inhibition of fear learning is also very different in infancy than in adulthood. Specifically, extinction of fear early in life is much more resistant to relapse than is extinction later in life. Both of these findings suggest that young animals should be especially resilient to the emergence of mental health disorders, which appears to be at odds with the view that early-life experiences are particularly important for the development of later psychopathologies (such as anxiety disorders) and with the finding that the majority of anxiety disorders first emerge in adolescence or childhood. This apparent paradox might be resolved, however, if exposure to chronic stress early in life affects the maturation of the fear retention and extinction systems, leading to a faster transition to the adult form of each (i.e., long-lasting fear memories and relapse-prone extinction). In several recent studies we have found exactly this pattern; that is, infant rats exposed to maternal-separation stress exhibit adult-like fear and extinction learning early in development. Further, we have demonstrated that some of these effects can be mimicked by exposing the mother to the stress hormone corticosterone in their drinking water (in lieu of the separation procedure). These findings suggest that early-life exposure to stress and stress hormones may act as a general signal that can alter the developmental trajectory of emotional systems and potentially place animals at greater risk for the development of anxiety. The implications of these recent findings for our understanding of the developmental origins of health and disease, and for enhancing preventative and therapeutic treatments across the lifespan, are considered.

## Review

Early-life has long been considered a critical time period for the establishment of one’s mental health trajectory. Childhood or adolescence is the typical age of onset for many mental health problems [1,2] and numerous theories regard early experiences as the foundation for life-long mental health function [3-6]. This perception of early life as a critical period for later emotional functioning has prompted the suggestion that prevention and treatment for mental health disorders be targeted to the early years [1,2]. Despite the proposed importance of early-development in establishing mental health, the empirical research examining emotional functioning has largely focused on adults, with this being especially true for animal models [7]. A greater appreciation of how emotional learning develops across the lifespan is likely to yield insights into how to best manipulate these forms of learning at different stages of development in order to either prevent the emergence of mental health disorders or effectively treat such disorders should they occur.

Primarily because of their potential for translation, two animal models of emotional learning have received considerable attention, namely, the ability to learn about fear (e.g., through Pavlovian conditioning) and the ability to inhibit learned fear associations (e.g., through the process of extinction). Not only are the neural structures which support these forms of learning similar in rodents and humans, anxiety disorders in humans are proposed to emerge when these processes are dysregulated [8-11]. For example, individuals with panic disorder have been shown to exhibit a memory bias for threat-related information and individuals with post-traumatic stress disorder (PTSD) exhibit deficits in their ability to inhibit fear [8-10,12]. Further, the most widely employed and empirically validated treatment for anxiety disorders in adult humans is exposure therapy, which is modeled upon the process of fear extinction [13]. Interestingly, studies which have examined these processes developmentally have consistently shown that fear conditioning and fear extinction operate very differently in infancy than in adulthood. Further, the observed developmental differences in fear learning and fear extinction challenge the idea that early life is a critical period for the establishment of mental health disorders. Specifically, infant rats exhibit profoundly faster rates of forgetting (i.e., infantile amnesia) than do adults [14] and more recent research has shown that when extinction occurs early in life relapse is much less likely to happen than when extinction takes place later in life [15]. Both of these findings suggest that young animals should be especially resilient to emergence of mental health disorders, which would appear to be at odds with the notion that early-life experiences are especially critical for establishing one’s mental health trajectory.

While the developmental findings just described seem to be contradictory to the long-held view that one’s mental health is profoundly affected by early-life experiences, more recent research from our laboratory suggests a solution to this apparent discrepancy. In particular, we have shown that the maturation of the fear memory and extinction systems is regulated by the rearing environment of an animal, leading rats with a history of stressful rearing conditions to transition more rapidly into the adult fear conditioning and extinction systems (i.e., infants from an adverse rearing environment exhibit long-lasting fear memories and relapse-prone extinction). Taken together with other research on the effects of stress on emotional learning, these findings suggest that early-life exposure to stress and stress hormones may act as a general signal that can alter the developmental trajectory of emotional systems and potentially place an individual at greater risk for the development of anxiety. Considering the potential relevance of these findings for our conception of early vulnerability and resilience, this review summarizes these recent advances in our understanding of the development of emotional learning following stress.

### Fear and extinction learning in the developing rat

The processes of fear expression and fear extinction in adult rats have been the focus of much research over the past few decades, leading to what is now a good understanding of the neural mechanisms and behavioral consequences of these forms of learning in the adult. For example, it is well known that adult animals are particularly apt at learning about and retaining memories of conditioned fear associations over periods of many weeks to months e.g., [16]. In addition, adults are known to use an extinction system which is susceptible to fear relapse; after extinction training, conditioned fear can return in adult rats if they are tested in a different context (fear renewal), are given a brief reminder treatment (e.g., a foot shock; fear reinstatement), or merely following the passage of time (spontaneous recovery of fear; [17]). On the other hand, fear conditioning and extinction in infants appears to involve very different mechanisms, resulting in very different behavioral outcomes.

One of the major developmental differences in emotional learning concerns the poorer retention abilities of infants compared to adults, a phenomenon known as *infantile amnesia*. In one of the earliest studies examining infantile amnesia in non-human animals, rats trained at different ages from between postnatal day (P) 18 and P100 were shown to be equally apt at forming an association between the black side of a black-white shuttle box and footshock, as assessed by passive avoidance of the black side when tested immediately after training. However, after a training-test interval of one week P18 rats were shown to forget, exhibiting reduced latencies to enter the black side of the shuttle box. The adult rats, in contrast, exhibited excellent retention even when tested as long as 42 days after training [14]. These findings suggest that the ability of animals to retain lasting memories of events is slow to develop.

Another feature of emotional learning that changes across development is the tendency to exhibit relapse-resistant fear extinction, which has recently been shown to occur only during a brief postnatal window [i.e., approximately between P16-P21; see 15 for a review of this work]. Briefly, in those studies, rats trained on P16 (i.e., during infancy) and extinguished the following day did not exhibit renewal or reinstatement of conditioned fear. In other words, fear was permanently inhibited in infant rats following extinction training. In contrast, if rats were trained just one week later (at P23; the juvenile period of development in the rat) they exhibited adult-like extinction, characterized by high return of fear in the renewal and reinstatement preparations [18-20]. More recently, the absence of the renewal effect was replicated in infant mice [21]. Further, that study showed that when mice were tested 10 days post-extinction those that had been extinguished at P24 exhibited spontaneous recovery while those extinguished at P17 maintained low levels of freezing (i.e., they did not exhibit spontaneous recovery of the learned fear). Together these studies suggest that the tendency to exhibit fear relapse following extinction training also emerges relatively late in postnatal development (i.e., in the juvenile period).

These findings on infant fear conditioning and extinction would seem to be at odds with the epidemiological data implicating early life as a critical period for the emergence of mental health problems. Implicit in the view that early experiences play a particularly critical role in the development of adult mental health disorders is that these individuals retain and retrieve those early experiences across development [22]. However, the data just reviewed suggests that, at least under normal laboratory rearing environments, infant rats rapidly forget aversive events and are able to effectively and permanently inhibit fear responses. Hence, these empirical data suggest that the young should be relatively protected from the development of anxiety. One potential solution to this apparent inconsistency would be that the vulnerability typically associated with early life experiences may occur only under specific conditions. In other words, perhaps infant rats exhibit good fear retention and relapse of fear after extinction training when they have been reared in environments where such behaviors may favor survival (i.e., under conditions of adversity).

### The regulation of fear and extinction learning by adverse early-life experiences

One experience that has been consistently associated with increased risk of poor mental health outcomes is childhood adversity, suggesting that early stressful experiences may affect the development of emotion regulation systems and predispose individuals to mental health problems. For example, exposure to a range of childhood adversities such as maladaptive family functioning (e.g., parental mental illness, physical abuse, neglect, parental criminality) and trauma exposure have been shown to substantially increase the risk of onset (and to a lesser degree, persistence) for most mental health problems, with exposure to multiple traumas having an additive effect of disorder likelihood [23,24]. Interestingly, both rats and humans are known to experience a ‘stress hypo-responsive period’ (SHRP) early in life, during which time the hypothalamic pituitary adrenal (HPA) axis is relatively quiescent and circulating corticosterone/cortisol levels are low [25,26]. The occurrence of a SHRP suggests that high levels of stress hormones are disruptive to the normative development of the brain and could therefore affect the maturation of behaviors which are dependent on those brain systems that are normally developing at the time of exposure (e.g., emotional learning systems). Indeed, there are several examples in the literature showing that exposure to stress at a time when corticosterone levels are normally low results in early transitions between infant- and adult-like forms of emotional learning. For example, in the second postnatal week of life rats experience a developmental transition in their behavioral and neural response to an odor paired with shock. Specifically, in rats aged P10 and older odor-shock conditioning leads to subsequent avoidance of the odor. However, this avoidance response is not displayed when rats are conditioned at P6-8. Rather, rats conditioned at the younger age exhibit a paradoxical approach response towards the odor [27]. In addition, while odor-shock conditioning activates the amygdala in rats aged P10 and older, the same conditioning procedure has no effect on amygdala activity in P8 rats [28], suggesting that different neural structures are involved in the conditioned responses exhibited by P10 and P8 rats. Interestingly, if rats were raised in a stressful rearing environment, or were given a corticosterone injection before test, then a precocious avoidance response to the shock paired odor was observed at P8, which was correlated with increased amygdala activity [28-32]. These studies show that the way in which an animal responds to cued fear associations depends not only on its chronological age but also on its early life experiences.

Accelerated transitions following early stress or HPA axis activation also occur in humans. For example, typically developing children exhibit enhanced amygdala activity to neutral over fearful faces using functional magnetic resonance imaging (fMRI), whereas adults show the opposite pattern of activity (i.e., enhanced amygdala activity to fearful over neutral faces; [33]). In studies in which researchers compared typically developing children to children that had been previously institutionalized (a naturally occurring model of infant neglect), it was shown that the previously institutionalized children exhibited the adult-like pattern of amygdala response to fearful and neutral faces, suggesting that amygdala development had been accelerated in these children [34]. It is interesting also to note that early exposure to glucocorticoids or stress has been shown to accelerate some aspects of amygdala development in animal models. For example, rats that were exposed to the stress of early weaning (i.e., at P14 rather than at around P23 as occurs standardly) exhibited accelerated myelination specifically in the basolateral amygdala [35]. The cross-species convergence of these findings further highlights the potential usefulness of animal models in understanding the effect of stress on the early development of emotional systems.

In the studies just described, premature stimulation of the HPA axis led to the early onset of behaviors which were typical of older animals. Another possibility then is that early stimulation of the HPA axis (either through the application of a stressor or through administration of stress hormones, e.g., corticosterone) will cause an early transition between infant and adult-like forms of fear retention and extinction learning. That is, premature stimulation of the HPA axis may act as a general trigger for the transition from immature to mature forms of emotional learning. To investigate this possibility, we recently examined whether the age at which rats begin to display adult-like forms of fear retention and extinction can be manipulated by exposing them to early-life stress.

In the first study to investigate the role of chronic stress on extinction behaviors in the infant rat, we [36] exposed rats to repeated bouts of maternal-separation (MS; 3 hours per day from P2-14) and then tested these animals for the occurrence of various relapse-related phenomena after extinction training on P17. The MS animals were compared to a standard-reared (SR) group of infant rats. While SR P17 rats exhibited the typical infant profile of extinction (i.e., they did not show renewal or reinstatement effects), MS P17 rats behaved more like adults. In other words, after extinction training in infancy MS rats exhibited renewal and reinstatement effects. In addition, the maternally-separated rats used a neurotransmitter (gamma amino butyric acid; GABA) during extinction learning that is not involved in the infant extinction system but that is involved in the adult extinction system. In a follow-up study it was shown that maternal-separation also accelerated the transition into and out of adolescent extinction behaviors [37]. Specifically, past research has shown that extinction training during adolescence is characterized by decreased involvement of the prefrontal cortex during extinction learning and poorer retention of extinction training [38,39]. However, after maternal-separation stress rats begin to exhibit the adolescent profile of poor extinction retention earlier and this profile ended earlier also [37]. Together, these studies show that maternal-separation stress appears to lead to a leftward shift in the developmental trajectory of the adolescent and adult-like fear extinction systems, allowing these systems to come online earlier in development than is typically seen in non-stressed rats. Clinically, these studies suggest that the propensity for young individuals to exhibit relapse following extinction training may differ as a function of their early-life rearing experiences, with those who have experienced stress being prone to relapse earlier in development.

It is not only the trajectory of the extinction system that is accelerated by early-life stress. Recently, we reported that the development of fear retention is influenced by early-life stress [40]. In those experiments rats were exposed to the same adverse rearing conditions just described (MS) or were standard-reared. Then, at P17 rats were trained and tested for their retention of fear either 1 day later (when fear memories formed in infancy are usually expressed) or 10 days later (when infantile amnesia normally occurs). As expected, infant rats exposed to SR conditions exhibited good fear retention at the 1 day interval but had forgotten by the 10 day interval. MS infant rats, on the other hand, expressed excellent fear retention at both the 1 and 10 day retention interval. In addition, it was shown that those MS rats conditioned on P17 remembered for up to 30 days post-training when compared to an unpaired control group of MS infants. To further understand this effect, we then examined whether animals had to be exposed to MS or whether maternal exposure to stress hormones was sufficient to cause an early transition in fear retention. To answer that question, rather than going through the maternal separation procedure animals were reared by mothers given either corticosterone- or vehicle-supplemented drinking water across the same period of time as MS (i.e., P2-14). Then, P17 pups were trained in the same way as in the earlier experiments. It was found that pups nursed by the corticosterone-treated dams (CORT-nursed) showed the same early transition as the MS pups from the earlier studies [40]. That is, while the pups nursed by vehicle-treated dams forgot a fear association formed on P17 within a 10-day period, the CORT-nursed pups remembered over this time. Hence, in addition to exhibiting greater relapse after extinction training, the studies just described demonstrated that MS and other early-life stressors (dam treatment with CORT) also led infant rats to retain fear associations for longer periods of time (see Table 1 for a summary of these results).

**Table 1:** Summary of the behavioral characteristics of the adult-and infant-like fear and extinction systems, along with the effect of stress on the characteristics of the infant system

	**Juvenile and adult**	**Infant**	**Maternally-separated infant**
Renewal	✓	***x***	✓
Reinstatement	✓	***x***	✓
GABA involvement in extinction	✓	***x***	✓
Infantile amnesia	***x***	✓	***x***

Taken together, the studies reviewed above suggest that the first few weeks of life in the rodent represent a ‘critical period’ for the development of emotion regulation behaviors. Specifically, many forms of emotional learning which are typical of adults emerge around the end of the SHRP and can be prematurely stimulated by stress or direct application of glucocorticoids. These findings suggest that stress and stress hormones may act as a general developmental switch, stimulating early transitions across a variety of systems responsible for different aspects of emotional learning.

## Conclusions

While preclinical models of infantile amnesia and extinction across development were first investigated nearly 50 years ago, until very recently these phenomena had not been examined in the context of early-life stress. Considering the proposed importance of infant fear memories for psychiatric functioning [4-6], and the high degree of comorbidity between psychiatric disorders and specific early-life experiences (e.g., adversity [41]), understanding the influence of stress on these forms of emotional learning is a clinically important question. The studies described in this review highlight the dynamic nature of emotional system development across the early postnatal period and show that the maturation of these systems in rats is experience-dependent. In addition, within the human literature, there are numerous reports of individual differences in the processes of fear retention and extinction which may underlie subsequent vulnerability to develop anxiety disorders e.g., [42,43], yet there is little information on how these individual differences might emerge. The work just reviewed may provide some insight into individual differences in vulnerability to mental health problems and how early-life adversity may lead to the emergence of anxiety disorders as they demonstrate that infant rats exposed to early adversity exhibit better retention of fear across long periods of time and greater relapse after extinction than their non-stressed peers.

There are multiple candidate mechanisms which could potentially work alone, or in concert, to produce the behavioral outcomes on fear and extinction learning we observe following maternal-separation. One possibility is that maternal-separation might alter the maternal behavior that mothers exhibit towards their pups and this affects maturation of the pups’ emotional systems. Indeed, maternal-separation has been reported to alter maternal behaviors exhibited by dams, such as increasing ‘active’ maternal behaviors (e.g., arched back nursing and pup licking and grooming) in the hours immediately after the pups are reunited with the mother [44-46]. However, some studies have shown a dissociation in the effects of maternal-separation treatments on maternal care and stress-response and fear outcomes in the offspring [44]. In that study total levels of maternal care were indistinguishable between litters exposed to maternal-separation or early-handling manipulations but the two procedures still produced different outcomes on fear behavior and stress responses in the adult offspring. It is unclear at present whether the alterations in pup emotional system maturation that we have observed are due to differences in maternal behavior following the maternal separation procedure. Another possible mediator of the effects of MS on pup fear retention and extinction behaviors is that maternal-separation may have resulted in some epigenetic alterations to gene promoters in the pups or mothers which are involved in regulation of the stress response or neural maturation. For example, a recent study showed that maternal-separation produced a stable increase in the expression of two micro RNA (miR), i.e., miR 132 and miR 124, in the mouse PFC which was evident at P14 and in adulthood [47]. Those specific miR are known to regulate mRNA transcripts critical for brain development (e.g., neuronal morphogenesis and differentiation; [48,49]). Further, gene promoters for those miR contain a CpG island [49], which can act as a site of epigenetic modification of the gene promoter (e.g., DNA methylation). Hence, MS-induced epigenetic changes to gene promoters for miR 132, miR 124, or other mRNA involved in neural development might underlie the behavioral effects of accelerated emotional maturation described here. If so, the stable changes in gene expression caused by MS are likely to result in altered maturation of emotion systems across the lifespan, an end product of which may be accelerated ageing, a heretofore underexplored area. The possibility that epigenetic alterations and changes in maternal behavior contribute to the emotion learning outcomes we see after maternal-separation should be the focus of future studies.

While the reviewed studies are clearly important in furthering our understanding of the early-emerging manifestations of stress exposure on emotional learning, these studies also have the potential for furthering our understanding of adult mental health and how that might be treated. Specifically, the mechanisms via which early-life stress/corticosterone-exposure accelerated the emergence of adult-like fear retention and extinction might help to uncover mechanisms by which adult-like fear and extinction memories can be made to look ‘infant-like’ again. Indeed, it would be valuable clinically to understand how rapid forgetting of fear memories and relapse-resistant extinction could be promoted in the adult. By understanding what cellular and molecular mechanisms are involved in infantile amnesia and expression of infant-like extinction it may be possible to pharmacologically manipulate these in the adult to reinstate these behaviors. For example, it may be possible to switch on the infant retention and extinction systems in the adult to decrease retrieval of adult fear memories or decrease the chance of relapse following an extinction treatment. Although some success has been made in reactivating infant-like extinction in adult rats [21], no one has yet examined whether infant-like forgetting can be reactivated in the adult. In sum, understanding the development of emotional learning, and which factors can derail normative development, may result in enhanced treatments for anxiety which can be applied across the lifespan.

## Abbreviations

CORT: Corticosterone; fMRI: Functional magnetic resonance imaging; GABA: Gamma-aminobutyric acid; HPA: Hypothalamic-pituitary-adrenal; MS: Maternal-separation; P: Postnatal day; PTSD: Post-traumatic stress disorder; SHRP: Stress Hypo-responsive Period; SR: Standard-reared.

## Competing interests

The authors declare that they have no competing interests.

## Authors’ contributions

BC and RR conceptualized the manuscript, and then BC wrote the first draft. Both authors worked on subsequent versions of the manuscript, and both approved the final manuscript.

## Authors’ information

BC is a postdoctoral researcher and registered Psychologist working at The University of New South Wales, Australia. RR is a Professor in Psychology at The University of New South Wales.
